# Dosimetric comparison between coplanar and non coplanar field radiotherapy for ethmoid sinus cancer

**DOI:** 10.1186/1748-717X-2-35

**Published:** 2007-09-18

**Authors:** Antoine Serre, Katia Idri, Pascal Fenoglietto, Norbert Ailleres, Lore Santoro, Claire Lemanski, Renaud Garrel, Marc Makeieff, Ali Allaw, Jean-Bernard Dubois, David Azria

**Affiliations:** 1 Department of Radiation Oncology, Val d'Aurelle Cancer Institute, Montpellier, France; 2 Radiophysics Unit, Val d'Aurelle Cancer Institute, Montpellier, France; 3 Department of Head and Neck Surgery, University Hospital Gui De Chauliac, Montpellier, France

## Abstract

**Background:**

To compare non coplanar field (NCF) with coplanar field (CF) -intensity-modulated radiotherapy (IMRT) planning for ethmoid cancer.

**Methods:**

Seven patients treated with NCF IMRT for ethmoid cancer were studied. A CF IMRT optimization was prepared with the same constraints as for the NCF treatment. The maximum point doses (D max) obtained for the different optic pathway structures (OPS) should differ no more than 3% from those achieved with the NCF IMRT plan. The distribution of the dose in the target volume and in the critical structures was compared between the two techniques, as well as the Conformity (CI) and the Homogeneity Indexes (HI) in the target volume.

**Results:**

We noted no difference between the two techniques in the OPS for the D1, D2, and D5%, in the inner ear and controlateral lens for the average Dmax, in the temporo-mandibular joints for the average mean dose, in the cord and brainstem for the average D1%. The dose-volume histograms were slightly better with the NCF treatment plan for the planning target volume (PTV) with a marginally better HI but no impact on CI. We found a great improvement in the PTV coverage with the CF treatment plan for two patients with T4 tumors.

**Conclusion:**

IMRT is one of the treatment options for ethmoid cancer. The PTV coverage is optimal without compromising the protection of the OPS. The impact of non coplanar versus coplanar set up is very slight.

## Background

Ethmoid sinus cancers are rare malignant tumors of the paranasal sinuses. They are often diagnosed at a late stage and are often, at that point, locally advanced. Despite the lack of randomised studies [1-3] we have a multidisciplinary approach with initial surgery and adjuvant radiotherapy. Planning the radiation treatment is a challenge for the radiophysician due to the proximity of critical and radiosensitive structures. The outcome is suboptimal with locoregional failure and treatment morbidity [4-9]. The implementation of intensity modulated radiotherapy (IMRT) for this pathology offers a better bilateral sparing of the optic pathways and probably increases the therapeutic ratio. Nevertheless, the coverage of the target volume depends on the dose delivered to optic structure [10]. In daily therapeutic practice, the physician has to make a decision based on the probabilities for locoregional control and the risks of loosing binocular vision. The possible contribution of non coplanar field (NCF) as opposed to coplanar field (CF) IMRT is not well known. In our hospital, 7 patients were treated for ethmoid sinus carcinoma by IMRT with a non coplanar technique. The dose delivered to the optic structures depended on initial staging of the pathology and was at the discretion of the physician. For all these patients, we came up with a coplanar treatment plan, with the same maximum doses to optic structures as those obtained with NCF. The aim of this paper was to compare the dose distribution in the target volumes and in other various critical structures in coplanar and non coplanar field IMRT.

## Methods

### Patients

Between July 2004 and April 2005, 7 consecutive patients (3 males and 4 females) with node-negative ethmoid sinus tumors (based on the CT scan) were treated in the radiotherapy department of the CRLC Val D'Aurelle – Paul Lamarque. The median age was 51.7 years old (range 24 to 72 years). The initial staging was performed clinically with a cervico-facial CT scan, a sinus MRI, a chest X-ray and an abdominal US scan. We detected three adenocarcinomas, three esthesioneuroblastomas and one undifferentiated neuroendocrine carcinoma. Four patients were treated with surgery and post operative radiotherapy, two patients with concomitant radio-chemotherapy and one with sequential radio-chemotherapy. The studied population consisted of three T2, two T3 and two T4; one with anterior orbital soft tissue invasion, the other with extensions in the cavernous sinus.

### Patient data acquisition

Patients were immobilized in supine position with a customized cushion (Moldcare, Bebig^®^) and a 5-point thermoplastic face mask. A CT scan (Picker PQ2000) with iodine injection was performed from the vertex to the sternum with 3 mm slice thickness spaced every 3 mm. Images were transferred to the virtual simulation system (Acqsim Philips) and the isocenter, located in the clinical target volume (CTV1), was defined directly after the CT scan acquisition. This referent point was marked on the patient face mask using mobile lasers.

### Contouring of target volumes

For non-operated patients, the gross tumor volume (GTV) was delineated after MRI-CT fusion scan. The CTV1 was defined with a 3D empiric margin of 4 mm around the GTV. For operated patients, the CTV1 was contoured after the same fusion scan with the help of the surgical report and the anatomo-pathologist's results. The CTV2 included all the CTV1, the ethmoid, the ipsilateral maxillary sinus, the nasal cavity, the sphenoid sinus and the caudal part of the frontal sinus. In the case of sphenoid invasion, the cavernous sinus was included and for orbit extension the whole orbit was delineated. The planning target volume (PTV) was determined by a 4 mm 3D margin around the CTV.

### Contouring of organs at risk

For all patients, the following structures were delineated: spinal cord, extended spinal cord (ext cord) made up of a 7 mm 3D margin, brainstem, frontal and parietal lobes, pituitary gland, optic chiasma, optic nerves, retinae, lenses increased by a 2 mm 3D safety margin, parotids, temporo-mandibular joints, lachrymal glands.

### Treatment protocol with non coplanar fields

The 7 patients were treated with 5 non coplanar fields: two fields with 95° and 265° gantry angulations without table rotation and three non coplanar fields with 35°, 320° and 345° gantry angulations with 90° table rotation.

Optimization was performed on the inverse planning system Eclipse Helios (Varian Medical Systems) with 6 MV photon beams. The dose constraints used for the different volumes are summarized in Table 1.

**Table 1:** Dose-volume constraint set used for inverse planning optimization

**Volume**	**Constraint**
PTV	V 95% > 95% prescribed dose
CTV	V 99% > 95% prescribed dose
Spinal cord	D max < 40 Gy
Ext cord	D max < 45 Gy
Brainstem	D max < 55 Gy
Frontal lob	D max < 60 Gy
Parietal lob	D max < 60 Gy
Hypophyse	D max < 55 Gy
Temporomandibular joints	D max < 60 Gy
Parotid	D mean < 26 Gy
	V 50% < 30 Gy
Lens	D max < 12 Gy
Optic nerve	D max < 55 Gy
Chiasma	D max < 55 Gy
Retina	D max < 55 Gy
Lachrymal glands	No constraint

The dose delivered to the optic pathways depended on the proximity of the CTV1. Therefore, to validate the treatment plan, the physician had to compromise between target volume coverage and the dose to the optic pathways.

### Comparative study using coplanar fields

The comparative study consisted of performing a treatment plan for each patient using coplanar fields with a maximum delivered dose (Dmax) to the chiasma and optic nerves within 3% of the Dmax delivered with the non coplanar treatment plan. Optimization was performed with the same inverse planning system using the set of constraints described in Table 1. Five 6 MV photon coplanar fields were applied to these patients with 0°, 70°, 140°, 210° and 290° gantry angulations, the latter being approved by the physician in charge of the patient.

The dose-volume histograms (DVH) were calculated for all the delineated volumes in the two different treatment plans. Conformity and homogeneity indices were calculated for the PTV1 [11]. Homogeneity index (HI) is defined by the difference between D1 and D99% divided by the prescribed dose. Conformity index (CI) is defined as follows:

CI = (TV/V_PTV_) × (TV/V_95%_)

TV: Treated Volume is the volume of PTV1 receiving the prescribed dose (95%)

V_PTV _is the volume of PTV

V_95% _is the volume enclosed in the isodose 95%

## Results

### Dose distribution in the optic pathways

**Figure 1:**
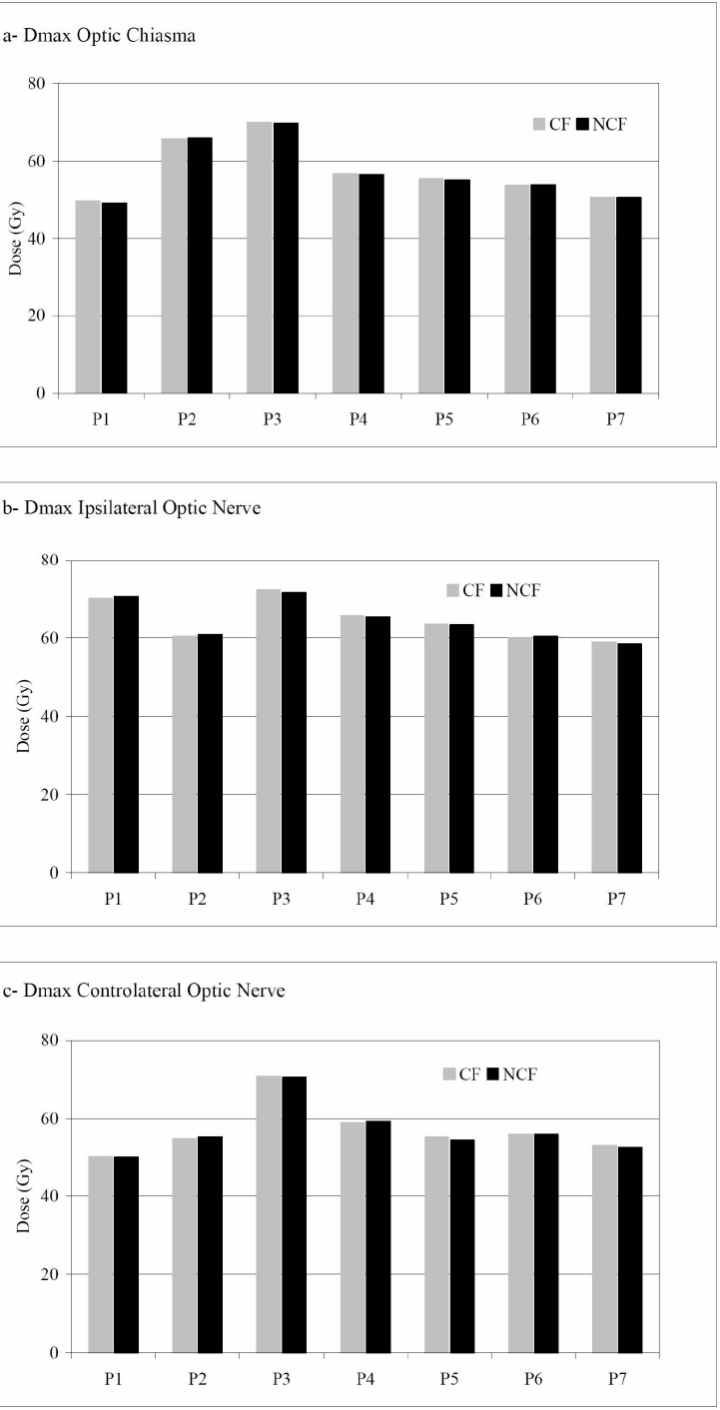
Maximum doses in optic pathways, respectively optic chiasma (a), ipsilateral optic nerve (b) and controlateral optic nerve (c). For each patient (Px), the maximum dose for coplanar field CF (grey) and for non coplanar field NCF (black) is represented.

The maximum dose in the optic chiasma, ipsilateral and controlateral optic nerves are represented in the Figure 1a,b,c. No difference was noted between NCF and CF techniques.

**Figure 2:**
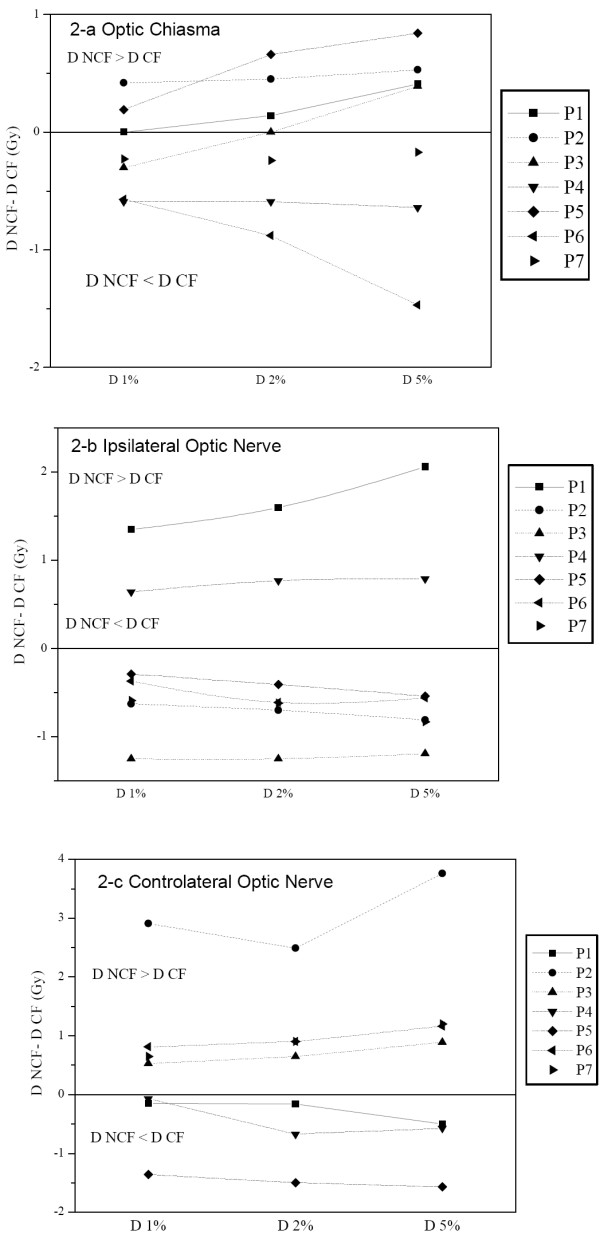
Dose distribution in optic pathways for each patient (Px); optic chiasma (a), ipsilateral optic nerve (b) and controlateral optic nerve (c) respectively. The dose difference in Gy between non coplanar field NCF and coplanar field CF (D NCF – D CF) is represented in terms of D1%, D2%, and D5% corresponding to the doses in 1, 2 and 5 % of the volumes respectively. This means that when the difference is negative, the dose to optic pathways is higher when using coplanar field technique.

Likewise, the doses delivered in 1, 2 and 5% of these volumes (D1, D2 and D5%) were similar for the two techniques (Figure 2a,b,c). The differences did not exceed 2 Gy except for patient n° 2, where the dose in the controlateral optic nerve was more than 3 Gy lower with the CF technique.

### Dose distribution in organs at risk

**Figure 3:**
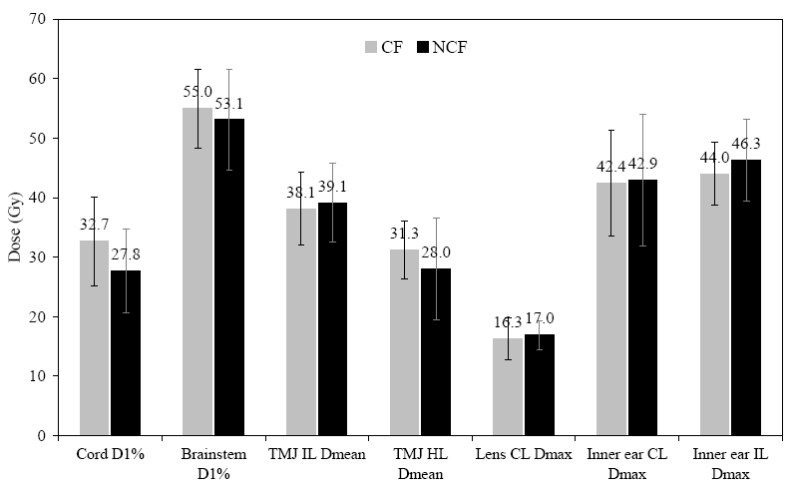
Comparison of dose distribution in organs at risk for the coplanar field technique CF (grey) and non coplanar field technique NCF (black). The most characteristic tolerance dose for each organ at risk is represented. D1%, Dmean and Dmax are the 1% of organ volume doses, mean dose and maximum dose respectively. Mean dose values and standard deviation are shown.

In Figure 3, we show the most characteristic tolerance doses for each organ at risk (OAR). Also represented are the mean values for each organ with their standard deviation. No difference was noted between CF and NCF for the ipsilateral and controlateral lenses, brainstem, temporo-mandibular joints, inner ears or parotids. Moreover, we did not find a subgroup of patients who benefited from either of the techniques.

### Dose distribution in the target volume

**Figure 4:**
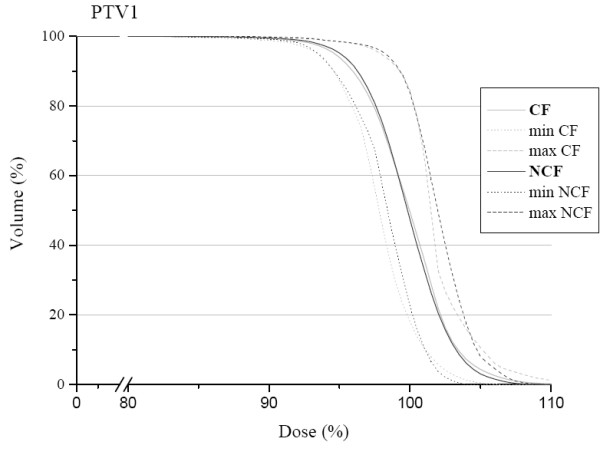
Mean dose volume histogram in the planning target volume for the coplanar field CF (grey) and non coplanar field technique NCF (black). Minimum and maximum doses of the study group are represented by dotted lines.

**Figure 5:**
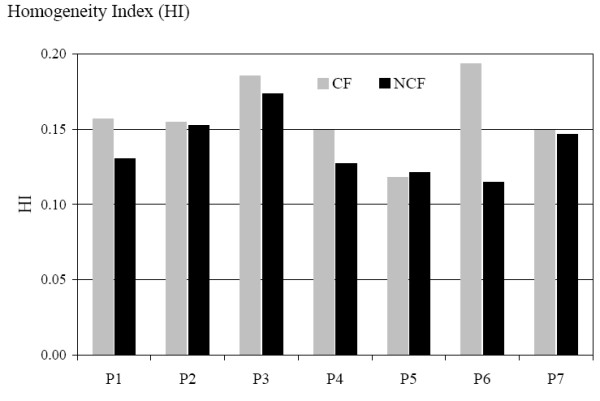
Homogeneity index (HI) for each patient in the coplanar field CF (grey) and non coplanar field NCF (black) technique. HI is defined as the difference between D1 and D99% divided by the prescribed dose. A perfect homogeneity would be reached with a zero index.

**Figure 6:**
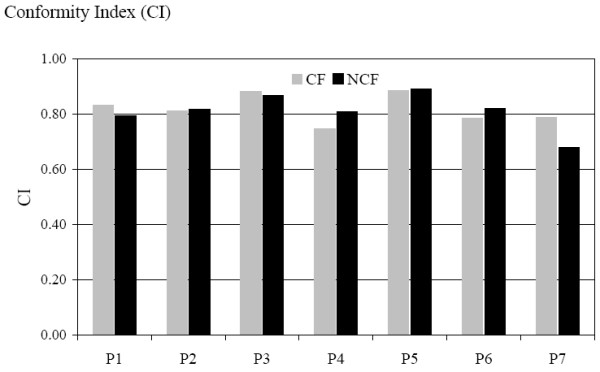
Conformal index CI for each patient in the coplanar field CF (grey) and non coplanar field NCF (black) technique. CI is defined as follows: CI = (TV/V_PTV_) × (TV/V_95%_) TV: Treated Volume is the volume of PTV1 receiving the prescribed dose (95%); V_PTV _is the volume of PTV; V_95% _is the volume enclosed in the isodose 95%

**Figure 7:**
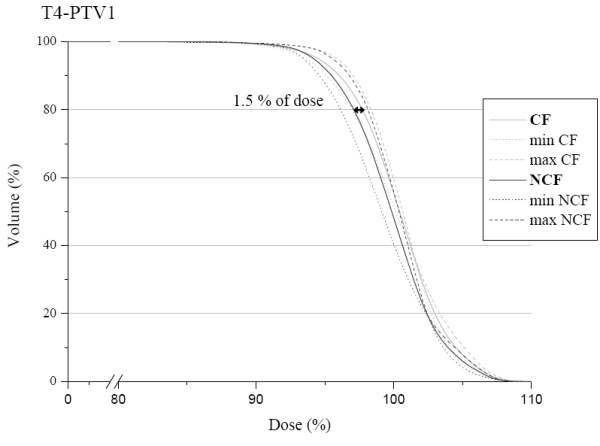
PTV mean dose volume histogram in the T4 subgroup for the coplanar field CF (grey) and non coplanar field technique NCF (black). Minimum and maximum doses of the study group are represented by dotted lines.

The mean dose volume histogram for the PTV1 is shown in Figure 4. We can observe a slight difference in favour of the NCF compared with the CF. The HI confirm this tendency with a mean value of 0.14 for the NCF and 0.16 for the CF; HIs are represented for each patient in Figure 5. The conformity index for the two techniques was similar as shown in Figure 6. In the subgroup of patients with skull base involvement (T4), a significant benefit was noticed with the CF compared to the NCF (Fig. 7). Ninety percent and 95% of the volume received respectively 96% and 94.5% of the prescribed dose with the CF technique and 94.5% and 93.5% with the NCF technique.

In the other subgroups, we observed no dosimetric impact of either of the techniques.

## Discussion

The implementation of modern radiotherapy (3D conformal and IMRT) has led to a reduction of the mean total dose to organs at risk and particularly to the optic nerves when compared to conventional radiotherapy [12]. Radiation optic neuropathy is highly dependant on the radiation dose [13-15]. Consequently, the high incidence of radiotherapy-induced blindness, as much as 37%, with conventional radiotherapy could be reduced by the use of 3D radiotherapy [16-18]. However in a single institution study with 40 patients [19], conformal radiotherapy for paranasal sinus carcinoma seems to be safer with only a 5 % incidence of cataract and only 2.5% unilateral blindness.

The benefits, in dose reduction to the optic pathways, of IMRT over 3D conformal radiotherapy have already been investigated [20], with an average maximum dose of 56.4 Gy for IMRT and of 64.2 Gy for 3D conformal radiotherapy. However, a complex treatment planning with 4-field 3D conformal radiotherapy and forward treatment planning will yield similar results to CF IMRT [21]. We have to point out though, that since high doses were delivered to both optic nerves, this diminishes the case for IMRT in this study.

One other advantage of IMRT is the mode of administration of radiotherapy: as integrated concomitant boosts with a daily dose fraction to OAR of less than 2 Gy which can prevent late complications [14].

In a publication by the M. D. Anderson Cancer Center, the maximum point dose for the controlateral optic nerve was significantly reduced when using 5 non coplanar field IMRT rather than the 9 coplanar field method [22]. On the other hand, there was no significant difference for the ipsilateral nerve. In our experience, we did not observe better D1, D2 and D5% to the optic pathways with the non coplanar fields when using the same maximum dose for both techniques.

The significance of maximum point dose to serial organs such as chiasma or optic nerves in fractionated radiotherapy is not well known, and many teams use D1 or D2 as the maximum tolerated dose for the validation of dosimetry. A Belgian study by Claus et al. introduced a planning organ at risk volume (PRV) made of a 2 mm isotropic expansion around the optic pathway with the following constraint: less than 5% of the PRV should receive more than 60 Gy [23]. A median follow up of 31 months [24] for the 39 patients did not show any radiotherapy-induced blindness. Moreover, a recent publication [25] with 36 patients treated by IMRT for paranasal carcinoma did not report decreased vision.

For small organ volumes such as the lens, we used a PRV with 2 mm isotropic margin. During optimization, constraints were modified in order to have the lowest dose possible. Maximum dose to the lens was very similar for both techniques, around 16 Gy. Probability of cataract remained high at 27% at 5 years and 57% at 8 years for a dose of 15 Gy in 15 fractions, as described by Henk et al. [26].

This excess dose to the lens does not depend on the radiotherapy technique, and even with conformal 3D treatment [12,21], the ipsilateral and controlateral lenses are irradiated more than the tolerated dose given by TD 5/5 published by Emami [27]. Proton therapy could be used to reduce the dose to the controlateral lens but 40% of the controlateral lens will still be overdosed [12], without any impact on the dose delivered to the ipsilateral lens.

It would be tempting to compare the two treatment modalities for the entire ocular globe. We know that coplanar IMRT and 3D conformal radiotherapy reduce the mean total dose in a similar way when compared to conventional irradiation [12], with a slight benefit with a 5-beam conformal radiotherapy over coplanar IMRT [28]. A significant dosimetric advantage represented by the mean dose is noted for non coplanar over coplanar field IMRT when considering the two ocular globes [22]. Nevertheless, the pertinence of these comparisons for clinical practice is doubtful because of the difference in radiosensibility for the diverse components of the eye [29].

The DVH and the conformity index for the PTV1 are similar between the two techniques. No available data demonstrating the superiority of the non coplanar over coplanar fields on the target volume has been published. This lack of difference can be explained by the ease with which the inverse planning system can follow the constraints prescribed for sliding window IMRT. No superiority of conformal 3D treatment over conventional treatment planning was demonstrated in maxillary sinus tumors [20]. In ethmoid carcinoma, conformal radiotherapy with forward planning is better than conventional planning [21,28].

In the publication of Adams et al., the conformity and homogeneity indices are better with IMRT than with conformal treatment [20]. The same observations were made when comparing multifield dynamic IMRT and step-and-shoot IMRT to 3D conformal treatment [30]. Interestingly, no dosimetric benefits for the target volume were noted with IMRT over conformal treatment planning in the maxillary sinus [12], probably due to the distance from the optic pathway and the lack of concave organs.

The MD Anderson Cancer Center experience did not directly compare coplanar and non coplanar field IMRT; however, they used a parallelized multi-resolution beam angle optimization (PMBAO) which included non coplanar fields and obtained thus better dose homogeneity without a real impact on the conformity index [22]. Their results could be explained by the use of only two non coplanar beams in a 5-beam configuration. The impact noted on the homogeneity in the target volume is in agreement with our results and seems to be due to non coplanar fields rather than the PMBAO.

## Conclusion

IMRT using the non coplanar field technique in ethmoid carcinoma is an effective approach for treating this tumor. A slight impact was shown on the PTV coverage for the non coplanar set up compared with the coplanar technique. Using our beam configuration, T4 tumors with skull base involvement were better treated with coplanar fields. In all cases, inverse planning allows for a dosimetric sparing of the optic pathways with good target volume coverage whatever the set up employed. The clinical impact on local control and on late effects is still not known with IMRT and a retrospective analysis of this cohort of patients is required.

## Abbreviations

IMRT- Intensity modulated radiotherapy;

NCF- Non coplanar field;

CF- Coplanar field;

GTV- Gross tumor volume;

CTV- Clinical target volume;

PTV- Planning target volume;

Dmax- Maximum delivered dose;

DVH- Dose-volume histogram;

HI- Homogeneity index;

OAR- Organ at risk;

PMBAO- Parallelized multi-resolution beam angle optimization.

## Competing interests

The author(s) declare that they have no competing interests.

## Authors' contributions

AS, KI, and PF conceived the study, collected data, and drafted the manuscript.

AA, LS, and NA collected data.

JBD and DA participated in coordination and helped to draft the manuscript.

CL, RG, and MM participated in the design of the study and assisted in data collection.

DA provided mentorship and edited the manuscript.

All authors have read and approved the final manuscript.
